# Moving in extreme environments: extreme loading; carriage versus distance

**DOI:** 10.1186/s13728-016-0047-z

**Published:** 2016-04-22

**Authors:** Samuel J. E. Lucas, Jørn W. Helge, Uwe H. W. Schütz, Ralph F. Goldman, James D. Cotter

**Affiliations:** School of Sport, Exercise and Rehabilitation Sciences, College of Life and Environmental Sciences, University of Birmingham, Birmingham, B15 2TT UK; Department of Physiology, University of Otago, Dunedin, New Zealand; Department of Biomedical Sciences, Faculty of Health Sciences, University of Copenhagen, Copenhagen, Denmark; Department of Diagnostic and Interventional Radiology, University Hospital of Ulm, Ulm, Germany; Orthopaedic Consulting Office at the Green Tower and Medical Pain Centre Lake Constance–Upper Swabia, Ravensburg, Germany; Comfort Technology, Inc., Hampton, USA; School of Physical Education, Sport and Exercise Sciences, University of Otago, Dunedin, New Zealand

**Keywords:** Ultra-endurance exercise, Load carriage, Environmental stress, Adaptation, Extreme loading/unloading, Fatigue

## Abstract

This review addresses human capacity for movement in the context of extreme loading and with it the combined effects of metabolic, biomechanical and gravitational stress on the human body. This topic encompasses extreme duration, as occurs in ultra-endurance competitions (e.g. adventure racing and transcontinental races) and expeditions (e.g. polar crossings), to the more gravitationally limited load carriage (e.g. in the military context). Juxtaposed to these circumstances is the extreme metabolic and mechanical unloading associated with space travel, prolonged bedrest and sedentary lifestyle, which may be at least as problematic, and are therefore included as a reference, e.g. when considering exposure, dangers and (mal)adaptations. As per the other reviews in this series, we describe the nature of the stress and the associated consequences; illustrate relevant regulations, including why and how they are set; present the pros and cons for self versus prescribed acute and chronic exposure; describe humans’ (mal)adaptations; and finally suggest future directions for practice and research. In summary, we describe adaptation patterns that are often U or J shaped and that over time minimal or no load carriage decreases the global load carrying capacity and eventually leads to severe adverse effects and manifest disease under minimal absolute but high relative loads. We advocate that further understanding of load carrying capacity and the inherent mechanisms leading to adverse effects may advantageously be studied in this perspective. With improved access to insightful and portable technologies, there are some exciting possibilities to explore these questions in this context.

## Background

This review within the series of *Moving in Extreme Environments* addresses human capacity for movement in the context of extreme loading and with it the combined effects of metabolic, biomechanical and gravitational stress on the human body. This topic encompasses extreme duration, as occurs in ultra-endurance competitions (e.g. adventure racing and transcontinental races) and expeditions (e.g. polar crossings), to the more gravitationally limited load carriage (e.g. in the military context). Because these circumstances overlap within themselves and with other reviews in this series, we discuss gravitational and energetic load within the ultra-endurance, expedition and occupational setting, leaving detailed discussion of related environmental factors on human tolerance and performance to those reviews—with the exception of cold-related effects since this is not discussed elsewhere. Juxtaposed to these circumstances is the extreme metabolic and mechanical unloading associated with space travel, prolonged bedrest and sedentary lifestyle, which may be at least as problematic, and are therefore included as a reference (e.g. when considering exposure, dangers and (mal)adaptations).

**Fig. 1:**
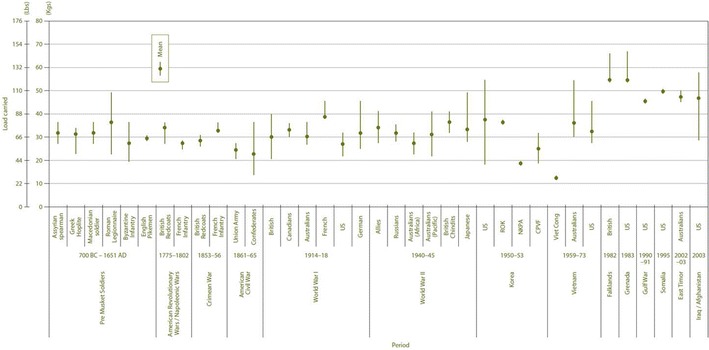
Historical representation of mean and ranges of carried loads by soldiers (reproduced with permission from [16])

Extreme loading pertains to the physical demands of carrying or towing mass, including or even exclusively oneself, as far or quickly as possible. The major resistive force is nearly always gravitational; hence the major stress is weight (Newtons, the product of mass and gravitational acceleration). Such stress impacts on all physiological systems. While the term ultra-endurance can describe exercise lasting more than just 4 h [1–3], our focus is on the more extreme end on this continuum, with exercise lasting many hours per day, over multiple consecutive days (e.g. >40-day Arctic expeditions [4, 5] or military training or operations [6–12]) or almost continuously for several days (e.g. adventure racing [13, 14]). Ultra-endurance competition might appear to be a relatively recent phenomenon, with—for example—the first adventure race being held in 1989 (Raid Gauloises), the first official 100-mile Western States Endurance Run in the United States of America held in 1977, the first Hawaii Ironman held in 1978 and, ~50 years earlier, the American Bunion Derby transcontinental foot races held in 1928 and 1929. The modern cycling Grand Tour stage races of Europe [i.e. Tour de France (first raced in 1903), Giro d’Italia (1909), Vuelta a España (1935)] have a longer history of challenging human capacities. All of these were preceded by the first long-distance cycle race in 1869 (L’Arc de Triomphe in Paris to the Cathedral in Rouen). Yet, load carriage in the military context and consideration of its impact on human capabilities has been an issue for many centuries (see [15, 16] and illustrated in Fig. 1). In addition, some modern ultra-endurance events/expeditions relive historical occupational tasks (esp. goods delivery before engine-based transport; e.g. Iditarod race [17]) and a form of ultra-endurance loading will be present in centuries-old spiritual pilgrimages as well as for the hunter–gatherer societies of past millennia. Indeed, endurance loading has shaped our genome and hence several important distinguishing features of our anatomy and physiology [18]. Perhaps, the earliest account of the consequences of extreme physiological loading is of Pheidippides, a hero of ancient Greece who reportedly collapsed and died after relaying the message of victory over Persia in the Battle of Marathon in 490 BC. Thus, the question of how the human body copes and responds to extreme feats of endurance has ancient origins and is still considered and challenged in the present day.

The purpose of this review, as with the others in this series, is to (1) describe the nature of the stress (i.e. extreme loading) and the associated dangers/consequences; (2) illustrate what, if any, regulations are in place as well as why and how they are set; (3) present the pros and cons for self versus prescribed acute and chronic exposure; (4) describe humans’ adaptation and/or maladaptation; and finally (5) suggest future directions for practice and research in this area.

## Review

### What is the stressor/danger for human movement?

Common to all the activities covered in this review is the requirement to carry or tow a load; at minimum an individual simply carrying themselves metabolically and mechanically against gravity, which can involve several vertical kilometres of ascent and descent. Additional load can be that carried in a backpack and webbing (ranging from a hydration system or survival equipment weighing <5 kg to large loads weighing >40 kg), towed in a sled (e.g. 120 kg [4] or 222 kg [5]), carried by hand (e.g. weapons or tools), worn as protection from environmental conditions or hostile elements (e.g. body armour, ~10 kg [19]) or some combination of these. The obvious consequence of this added load is the extra effort and physiological/physical cost (e.g. energetic, stress fractures, eccentric muscle damage) required to carry or tow it, which will be affected by the environmental conditions in which the work is undertaken. Indeed, these issues have been researched over several decades [e.g. [15, 20–22]], and reviewed accordingly [16, 19, 23–26]. Providing extensive detail on this is not within the scope of this review; however, there is a known additional cost of carrying more weight (e.g. [22, 27, 28]), which is lessened by carrying it closer to the centre of gravity (e.g. [23, 29]), thereby also lessening the additional perceived exertion [30]. The increased energy expenditure and physiological strain lowers work capacity, diminishes capabilities (although not necessarily generic to all physical tasks [31]), increases dietary requirements, increases heat stress (particularly if protective clothing is worn; see [32]), reduces mobility and potentially increases the risk of harm; ranging from musculoskeletal strains, to injury as a result of reduced cognitive performance associated with fatigue, through to fatality [e.g. carried loads of 27–41 kg attributed to many drownings during the D-Day landings at Omaha beach during World War II (see [15, 16])]. Yet, it is just as readily fatal to leave critical items in efforts to reduce the carried load, thus a trade-off between carrying essentials (e.g. food, clothing and weaponry) versus moving fast and effectively is fundamental in all of the situations discussed here: sport, occupation and military.

Illness and injury during extreme loading stress is an obvious danger related to this type of human endeavour. Fordham and colleagues reported that 73 % of their 223 adventure racing athletes reported musculoskeletal problems requiring them to stop training for at least 1 day, reduce training, take medicine or seek medical aid. We found a similarly high incidence of injury and illness; 38 of 48 athletes (79 %) reported a total of 49 musculoskeletal injuries during an adventure race [33]. Also prevalent in this 4- to 5-day near-continuous event were skin wounds and infections (43/49), upper respiratory illness (28/49) and gastrointestinal (GI) complaints (8/49; additional five 4-member teams withdrew due to GI complaints) [33]. One seemingly minor injury issue common to all extreme loading settings is the risk of repetitive rubbing on the locomotive limbs (usually of the feet and/or groin/thighs) and against items of carried load, developing into blistering and/or overuse injuries. Blistering and tissue degeneration can also accrue from intense or sustained exposure to heat, cold (see below) or water. While such injuries may have no more than a race-ending consequence in sport, in other settings, such as unsupported polar crossings or combat scenarios, the reduced capability and mobility and/or elevated risk of infection can have life-threatening consequences. Managing and preventing such injuries via optimising equipment (e.g. footwear, pack, body armour), reducing load and improving distribution are well recognised prevention actions for reducing the incidence of injury [23], but not always possible.

One environmental extreme mentioned briefly here is cold air exposure, because several features of prolonged exercise increase the risk of hypothermia and cold-related tissue injuries such as frost nip and frost bite. For example, polar expeditioning, cross-country ski racing, adventure racing and some military settings involve exposure to moderately dry or wet cold stress (e.g. in adventure racing [13]) through to extremely cold air (as low as −45 °C [5]), with only modest rates of heat production (see below). Cold stress is intensified by wind chill (see [34]), while some physical and physiological effects of cold stress are amplified by factors such as hypobaric hypoxia (e.g. elevation of 3000 m on the Polar plateau [5]), sleep deprivation and sustained energy deficit [12]. Prolonged exertion can impair cold tolerance by delaying the onset of shivering [10], impairing vasoconstrictive power of the exercised limbs [35], impairing thermogenic capacity [36, 37] and impairing dexterity and strength by at least 50 % even without core cooling [37, 38]. Yet, humans’ behavioural drive to minimise cold exposure is very strong [37], so their risk depends on their situation. Interestingly, whereas humans have strong adaptive responses to many aspects of prolonged loading (see below), little meaningful adaptation develops against cold exposure that would increase tolerance at the whole-body level [39, 40] or localised level [41], despite recent studies illustrating that some browning of adipose tissue can occur during repeated cold exposure, which would increase thermogenic capacity [42, 43]. Overall, the potential risks for human movement in cold air range from reduced strength and manual dexterity, to loss of mobility and function as a consequence of frost bite, to hypothermia-induced coma and subsequent death if the cold stress is not intervened.

**Fig. 2:**
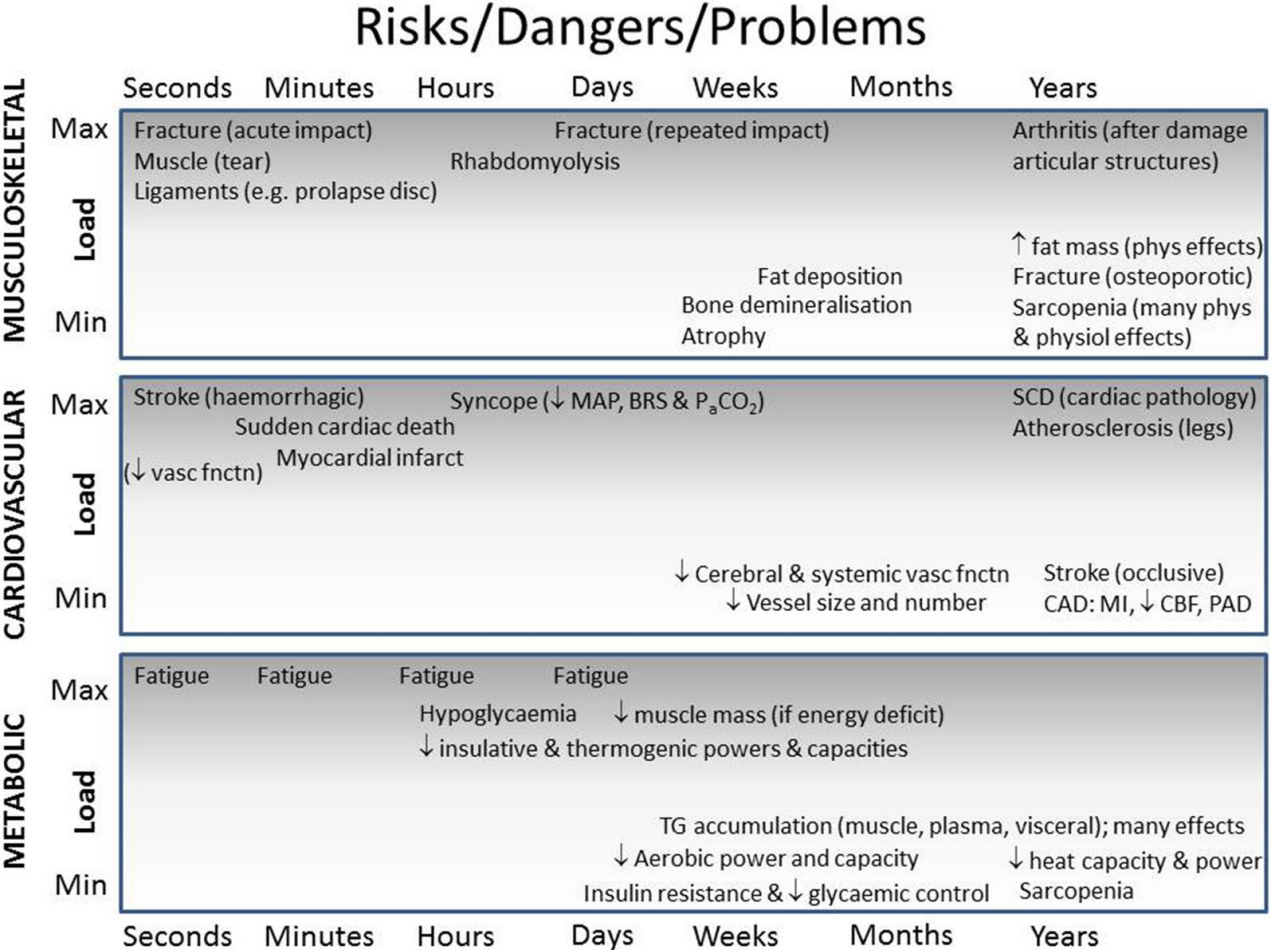
Illustration of adverse effects of the extremes of physical loading as a function of duration of exposure. *Phys* physical, *physiol* physiological, *MAP* mean arterial blood pressure, *BRS* baroreflex sensitivity, *PaCO*_*2*_ partial pressure of arterial carbon dioxide, *SCD* sudden cardiac death, *CAD* cardiac arterial disease, *MI* myocardial infarction, *CBF* cerebral blood flow, *PAD* peripheral arterial disease, *TG* triglycerides

In summary, all physiological systems are impacted by the prolonged metabolic and mechanical impacts of sustained loading, whether in sport, expeditioning or military settings. The consequences of such stress range from being little more than a nuisance to life threatening. These dangers should also be contextualised against those of the extreme unloading caused by sedentariness arising from bedrest, fear-avoidance behaviour due to chronic pain disorders or preferred behaviour. Figure 2 therefore summarises the consequences at both extremes of the spectrum of loading, within the physiological systems (different panels) and across exposure time. Within a few hours of ceasing movement, blood glucose regulation and endothelial function show impairment [44–46]. By 24 h, insulin desensitisation and loss of plasma volume also become evident. Even just reducing normal daily activity (steps) is enough to impair metabolic control and aerobic fitness [47]. These collective effects can ultimately be more debilitating, and make ‘physical inactivity’ the fourth largest contributor to early mortality in the world today [48]. The dangers of sedentary behaviour are thus becoming apparent as being both important and distinct from those of insufficient exercise, based on emerging evidence of its rapid-onset pathophysiological effects [46, 49] and on epidemiological evidence [50]. Importantly, unlike the high-load scenarios described above, the danger is that these effects are initially insidious and appeal to humans’ desire for comfort. Finally, it must also be acknowledged that the two extremes of loading can also be linked through loading-induced injury, causing immobilisation acutely through fracture, sprain or strain, or becoming chronic for or after many years of extreme loading (e.g. osteoarthritis). Thus, one danger of acute or chronic extreme loading is of consequent chronic unloading.

### What regulations are established, and why/how are they set?

Fatalities in the occupational or recreational setting often initiate reviews, discussion and/or an inquiry that then set new regulations and/or practice to minimise overt risk associated with extreme high-loading settings.

#### Ultra-endurance competition

The death of Nigel Aylott from a falling boulder dislodged by a fellow competitor in the 2004 Primal Quest adventure race highlights the risks and responsibilities that both racers and race organisers need to consider in conditions made extreme by both physiological (e.g. sleep deprivation, prolonged and continuous competitive exercise stress) and environmental factors inherent to such events (see [51]). Adventure races that are part of the Adventure Racing World Series have a set of *Rules of Competition* and a *Mandatory Equipment list* for safety purposes [52], e.g. team members must always be within 50 m of each other, each competitor must carry their own survival equipment and each team a communication device for emergencies. Additional items may be added by race organisers where they are specific to the location, conditions or laws of the host country. Technical competency requirements are also common (e.g. white-water or rope skills), and minimum experience standards may also be applied. Thus, the industry has provided its own regulatory standard, which is aligned with (and ultimately legally bound by) Occupational Health and Safety standards of the host country. Further, organisations such as the United States Adventure Racing Association have been established to guide and assist race directors and committees in conducting fun, safe and fair events [53].

For events like the Marathon des Sables (~6 marathons run in the desert over 6 days), race rules require competitors to hold down fluid or it will be given intravenously [54]. Interestingly, this ‘regulation’ comes with a time penalty, which certainly has the potential to create a negative perception and thus of appropriate and necessary treatment. A requirement of entry is medical certification of ones’ ability to participate, and a resting electrocardiogram report, both presented to the event’s medical team. Other requirements include ceasing forward travel during sandstorms.

#### Conditions during cold (arctic circle race)

In popular cross-country skiing competitions, temperatures below −25 °C on the major part of the course leads to race cancellation or delay, and with temperatures between −15 and −25 °C caution and specific information to participants on cold weather precautions is mandatory (see [55]). These temperatures are not uncommon in the Arctic Circle race in Greenland, and wind chill may lead to difficult race conditions especially when occurring on the cusp of the −25 °C postponement threshold. Race guidelines suggest that competitors should eat and drink whenever possible and every hour throughout the race. Such recommendations are intended to meet not only the increased energy and water-turnover requirements of the exercise (see below), but also of thermogenesis during exercise with cold stress [37].

To participate in this and other popular cross-country ski races, competitors must abide by the rules and regulations of the International Ski Federation (FIS, [56]) and hold a racer’s licence. Interestingly, the majority of the requirements to attain a racing licence from the FIS, and the rules that determine appropriate conduct as a licence holder, are mostly administrative and logistical (e.g. arriving at the correct time, overtaking protocol), while the health of competitors is deferred to National Associations. Thus, standardised and transparent criteria that need to be met for participation are not always clear.

In another extreme cold event, The Iditarod race (a 1000-mile sled race across Alaska [17]), competitors qualify via the *Muster Assessment* form, which is completed by judges and officials from other similar events. The assessment form considers ‘skills’ such as general attitude; ability to compete; physical stamina; cold weather preparedness and tolerance; compliance with race rules and policies; sleep deprivation tolerance; equipment selection; mental perseverance; organisation and efficiency; wilderness survival skills; and how an applicant treats their dogs. While this list is comprehensive in listing the potential stressors and behaviours that may be relevant to performance and survival, the ‘tick the box’ nature of the form again seems relatively subjective.

Overall, both the adventure racing and expedition/Nordic racing regulations seem light on rigour. However, perhaps the need to regulate these types of events is less since they will typically attract individuals looking to challenge themselves and have outdoor/wilderness experience and therefore will knowingly accept the responsibility and potential consequences. Yet, some duty of care should be expected of event organisers regardless of the experience and willingness of competitors to engage in such extreme events, as illustrated by the Nigel Aylott accident during the 2004 Primal Quest. Further, the lure of prize money (US$100,000 for winning that event) perhaps jeopardises racers’ safety to a larger extent than do the effects of sleep deprivation and environmental stressors. Ordinarily in ultra-endurance events, little such lure exists and it is both impossible and counter culture to remove all risks, so athletes who declare themselves experienced and aware of the disclosed risks (and agree to them via signed informed consent) must be expected to accept at least some responsibility for mishaps.

#### Military guidelines

The military has been a key player in setting industry standards for load carriage, especially in the heat. Guidelines have been set to determine the work-to-rest ratio and the amount of fluid consumed. These are determined by the exogenous thermal stress, assessed typically via the wet bulb-globe temperature index, the extent of physical exertion or load carried and other factors (esp. acclimatisation and protective clothing). The relevant research is reviewed extensively elsewhere (e.g. [57–59]), as are guidelines for operational procedures of acute and chronic protection of military personnel (e.g. [60–63]).

#### Sedentary activity, avoidance behaviour and bedrest

Chronic underloading is a danger with relatively high cost for quality of life, morbidity and mortality, faced by many more people in modern societies than are the settings mentioned above. It is also important to remember that such dangers are not annulled by regular exercise [50]. While exercise is recommended within the public health guidelines of many countries, and is mandated in educational curricula of some countries, regulations generally do not exist to either reduce sedentary behaviour or require asymptomatic people to undertake moderate vigorous physical activity, including exercise [64]. However, for chronic pain disorders (e.g. fibromyalgia, chronic low back pain) and in the rehabilitation phase after injuries on the musculoskeletal system, treatment standards are increasingly being established by national and international medical societies to prevent secondary disabilities or ongoing chronification caused by inappropriate and prolonged immobilisation or unconscious protection [65, 66]. Similarly, cardiac rehabilitation guidelines now include exercise training recommendations rather than bedrest, with exercise-based rehabilitation shown to reduce total mortality, cardiac mortality and hospital readmissions [67]. Ironically, this treatment strategy for cardiac rehabilitation is also a primary prevention for the original disease.

### Pros and cons of self vs prescribed exposure

Multiday adventure racing provides perhaps the upper limit of sustained loading acutely, with race competitors exercising nearly continuously across 3–10 days with very limited sleep (e.g. 1 + h/d). While there is certainly a potential for external pressure to continue exercising from fellow team members (often minimised by selection of team members of similar ability), such events provide a model to examine the upper limit of ‘self-prescribed’ exercise. The evidence to date indicates that homeostatic control of key regulated variables such as body core temperature and blood glucose levels is well maintained, despite the wide range of exercise intensities and ambient temperatures, and a large energy deficit [13, 68]. Thus, the prolonged and sustained nature of this acute exposure, along with the contributing effects of sleep deprivation in and of itself [69–71], would appear to be enough to counter the strong intrinsic motivation of athletes such that pace selection across the whole race remains appropriate to homeostatic requirements. Therefore, the need to impose regulations or restrictions does not seem necessary as physiological feedback mechanisms and changes in perception of exertion and reduced motivation as a consequence of sleep deprivation [69, 71] appear capable of protecting individuals from homeostatic failure. Recently, evidence of reduced central drive has been shown to occur during prolonged ultra-endurance exercise (110 km run [72]), providing more evidence for the ‘self’-preservation of homeostasis in this setting. Conversely, the high prevalence of non-steroidal anti-inflammatory drugs and analgesics use in these ultra-endurance athletes [33, 73], often taken alongside stimulants (e.g. caffeine) during competition to ward off the effects of the sleep deprivation, may have an impact on this homeostatic control. The net effect of such acute and chronic drug use on this type of performance and long-term health is unclear and requires further research [73].

Interestingly, the self-selected sustainable pace during these types of events (~40 % VO_2_ peak [13, 14]) is very similar to the work intensity (30–40 % VO_2_ max) maintained for multi-day military operations [74–77], and that predicted from laboratory-based work with varying carried loads for both males and females (~45 % VO_2_ max) [78]. These are obviously relative measures of aerobic power, therefore achieving optimal outcomes—whether in sport, military or other ultra-endurance tasks—requires distributing workload within the group so as to maximise effective velocity. Indeed, towing and load sharing is a common practice in adventure racing. However, the range of absolute aerobic capacities within a group may become an issue when the prescribed parameters of the task are not flexible, e.g. load sharing is not permitted or prudent. Historically, this is a classic scenario within a military training operation, where individuals are exposed to external (and internal; e.g. squad selection criteria) pressures to continue exercising and perform as instructed.

The ‘cons’ for self-prescribed acute exposure seem more relevant in shorter exposures, where strong intrinsic motivation has the potential to override physiological feedback. Indeed, the first 12 h of an adventure race is associated with more intense stress, in that competitors’ exercise pace far exceeds what is sustainable for the race [13, 14], perhaps reflecting a perception that giving up ground to other competitors early will impair the overall outcome, despite that being some days away. As such, the pure ‘self-prescribed’ pace in these early stages is somewhat influenced by other competitors and/or other external factors (e.g. dark zone regulations, whereby night travel is prohibited on some waterways) even among elite adventure racing athletes. An unresolved question—to our knowledge—is whether this asymmetric pacing is optimal in very prolonged endurance activity with or without substantive load carriage. Events such as iron man triathlon, single-day multisport events (e.g. New Zealand’s Coast to Coast race, >12 h) and multiday, stage events (e.g. grand cycle tours) show much higher intensities, typically around the anaerobic threshold (e.g. ~80 % [79–82]). It is in shorter periods like this that behaviour can compromise the effectiveness of physiological negative feedback loops and compromise homeostasis. Indeed, hypohydration and hyponatraemia have been reported during this type of ultra-endurance exercise [83] but are rare in longer events [13, 84–88], except perhaps hypoglycaemia during arm-dependent ultra-endurance exercise [68, 89]. Nevertheless, regardless of how motivated an individual is, the centralised control of homeostasis [90–92] will eventually prioritise survival if an organ’s nutrient or metabolite status is compromised (e.g. via fainting/collapse). The issue is how much strain is accrued on the way to that endpoint (e.g. of body core temperature, electrolyte content, endotoxic load, musculoskeletal trauma), and whether sufficient resources are available to recover homeostasis in any given environment.

Back at the other extreme, in the context of underloading caused by a sedentary lifestyle, clearly self-prescribed exposure is a global disaster, and one that is worsening as labour-saving devices and procedures develop further. While awareness of the benefits of regular physical activity is commonly acknowledged, including by people whose activity levels do not meet public health guidelines, awareness is lacking among the population as to differential effects of exercise vs. inactivity. As mentioned above, regular exercise does not cancel out the effects of sedentary behaviour [50], and this becomes more relevant in a built environment that seeks to reduce labour efforts and is not conducive to activity (e.g. removal of stairs for escalators, remote controlled devices etc.), removing potential opportunities for brief periods of activity/loading that can have positive effects on health [93]. Thus, both social and biological factors are mediating this epidemic of sedentary behaviour in the global population. This is why the biopsychosocial model has become a central strategy for physical and mental behavioural treatment of patients with chronic, geriatric and mental disorders in occupational, rehabilitation and pain medicine [94].

### What are the acute and adaptive and/or maladaptive responses to extreme loading?

#### Musculoskeletal

Depending on the nature of exposure, ultra-loading events may endanger the musculoskeletal system in different locations and ways. Because ultra-endurance races are based on the goal to complete a long distance on foot or non-motorised vehicles in general, the lower extremities are the main loaded parts of the human locomotion system. Until this century, little was known about the consequences of the ongoing biomechanical burden of ultra-endurance events on the bones, joints and soft tissues of the feet and legs. Even now most investigations on ultra-endurance events are limited to field studies on single events (adventure races, marathons, triathlons, bicycle, ski races, etc.) by relatively few researchers focusing on laboratory-based analyses, biomechanical measurements and non-criterion anthropometrical methods [95]. The diagnostic procedure of choice for endurance-related overuse injuries is magnetic resonance imaging (MRI) [96, 97], which provides a logistical challenge to implement in the field. Consequently, direct visualisation and analysis of biomechanical overuse reactions of the musculoskeletal tissues to ultra-endurance activity have not been investigated systematically until very recently. In 2009, the first (and still only) MRI field study was conducted in athletes completing a multistage ultra-endurance running event [the TransEurope FootRace project (TEFR project)]. Whilst following a large cohort of ultra-runners (n = 44) on their way across Europe (~4500 km and taking more than 64 days), a mobile MR-unit was utilised to obtain specific MRI data of overuse injuries [98]. The results of the TEFR project gave new insights on the adaptive possibilities and maladaptive responses of the lower extremity tissues to ultra-running loading. Key findings from this project illustrated how ultra-running impacts on the joints and cartilage, providing important objective data to contribute to the debate regarding risk or non-risk for development of arthrosis in the hip, knee or ankle joints [99, 100] and the circumstances leading to stress fractures.

The impact of prolonged repetitive stress on bone health is estimated via general rules and formulated propositions (Wolff’s law) [101]. Modern theories of bone remodelling predict the functional adaptation of the bone [102, 103], with its resilience to biomechanical impact depending on several individual factors including age, inherited material, preparation time (specific training), hormone status, sex, locomotive technique, peak load and location [104]. However, much less is known of the joint cartilage and its relationship with mechanical demand and biological adaptation. Serial quantitative MRI investigations of biochemical cartilage, as part of the TEFR project on hindfoot, ankle and knee joints, disproved any hypothesis or report that ongoing ultra-endurance running impact is harmful for healthy joints of lower extremities in the absence of obesity, proprioceptive deficit, poor muscle tone or malalignment [105, 106]. To the contrary, results indicated for the first time the ability of normal cartilage matrix to partially regenerate under ongoing multistage ultra-marathon burden in the ankle and hindfoot joints [98]. So, in general, running is joint protective [107, 108] and the magnitude of the distance where running might become dangerous for the joint tissues may be much further than has been previously predicted.

**Fig. 3:**
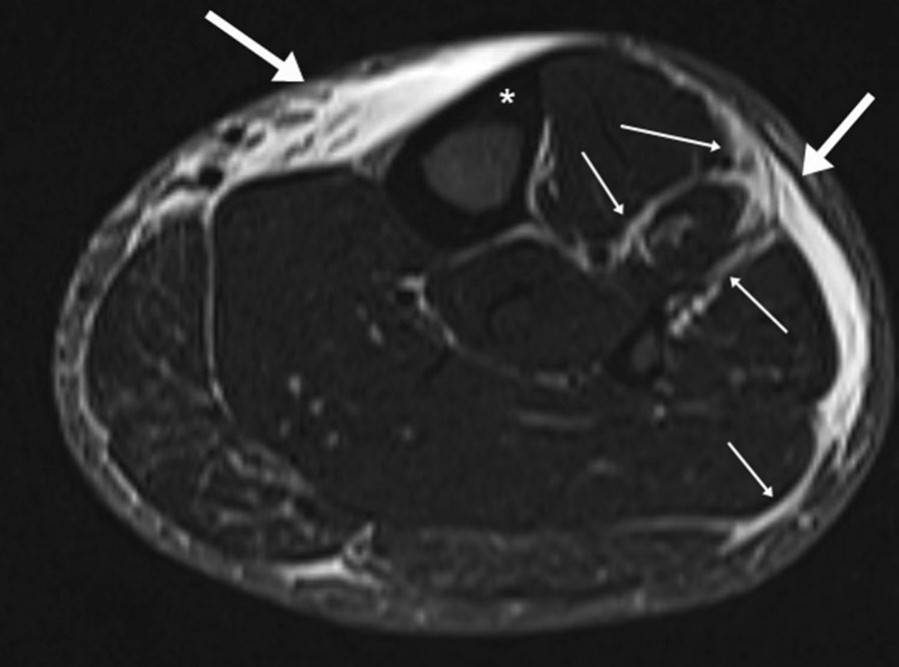
High water-sensitive MRI of the left lower leg (TIRM: turbo inversion recovery magnitude): severe “shin-splint” leading to premature termination of TEFR (47 years, male, stage 5 of TEFR, after 261-km run). *Thick arrow*: panniculitis, epifasciitis; *thin arrow*: myofasciitis and intermuscular fasciitis (extensors of the lower leg); * inert cortical bone (Tibia) without any periosteal bone reaction

The main reason for withdrawals in ultra-endurance competitions is overuse injuries of the soft tissues of the legs, mainly the tendons, muscles and fascia, summarised as the musculotendinous and myofascial system. Running specific terms like the shin-splint [109] and runner’s knee [110] are established for common overuse syndromes in the sport of endurance running [111, 112]. Their underlying pathophysiology is generally clarified. Specific mobile MRI of the legs in the TEFR project athletes showed that in ultra-running, overuse injuries are mainly intermuscular fascial inflammation processes beginning in one part of the leg. As detailed TEFR project images showed, the so-called shin-splint syndrome is mostly not associated with inflammation of the periosteum, as is commonly assumed, but only with myofascial inflammation of the extensors of the lower leg (see Fig. 3).

**Fig. 4:**
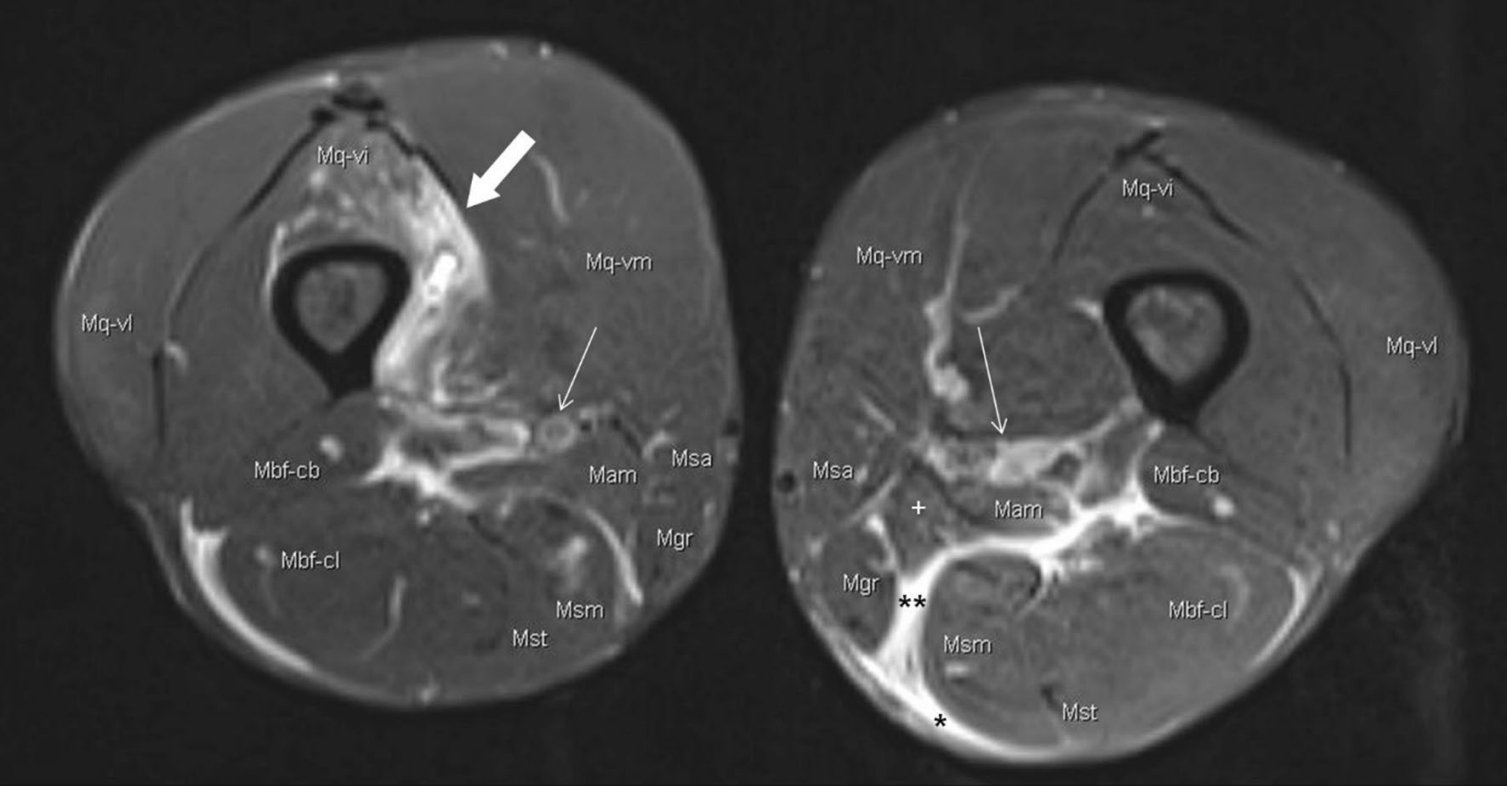
Water-sensitive MRI of upper legs (PDw: proton density weighting): muscle lesions and myofascial inflammation in the upper legs (56 years, male, stage 21 of TEFR, after 1521-km run). *Thick arrow*: muscle bundle rupture and myositis (M. quadriceps, Vastus intermedius); *thin arrow*: neurovascular bundle; * panniculitis, fasciitis; ** intermuscular fasciitis. *Mq* M. quadriceps, *-vl* vastus lateralis, *-vi* vastus intermedius, *-vm* vastus medialis, *Mam* M. adductor magnus, *Msa* M. sartorius, *Mgr* M. gracilis, *Msm* M. semimembranosus, *Mst* M. semitendinosus, *Mbf* M. biceps femoris, *-cl* caput longum, *-cb* caput brevis

These processes often expand via intermuscular fascial guide rails and lead to overuse problems in the same tissues of the contralateral leg due to asymmetric running when pain occurs in one leg. Pain-related cessation of running then becomes more likely. Figure 4 shows an example of such myofascial overuse problems in the upper legs of an experienced ultra-athlete from the TEFR. As myofascial and musculotendinous overuse injuries in ultra-endurance athletes often lead to withdrawal from a race, the pictured and many other cases from the TEFR show that they can mostly be overrun without any further tissue damage [98]. Nevertheless, a limit for the inflammation burden of these tissues will likely exist, therefore a functional compartment syndrome [113] as the endpoint of such processes has to be respected. Ongoing non-reduced loading may lead to fatal tissue necrosis and permanent damage [114]. Ensuring sufficient arterial and venous blood circulation is the basic prerequisite to overcome ultra-endurance burdens without further damage for the tissues, which is limited not only by the physical stress, but also by the environmental circumstances [115]. As a phylogenetic exception, the human foot seems to have a high resistance to mechanical impact even in the dimension of ultra-endurance loads, since relevant injuries are seldom observed or if they do then only in maladapted and untrained individuals [116–118].

Extrapolating these TEFR observations of musculoskeletal (mal)adaptations to other recently studied ultra-endurance events with extreme lower-limb loading (e.g. adventure racing and mountain ultra-marathon events such as the Tor-des-Geants) seems reasonable and relevant in two respects. First, such changes in the musculoskeletal system presumably contribute neural signals for pace selection [119]. Second, fatigue in such events appears to have a strong central component that develops relatively early and thus helps protect the musculoskeletal system. Evidence for such protection includes (i) direct measurement of neuromuscular fatigue before, during and after the Tor-des-Geants [120]; (ii) findings of equivalent fatigue in strength and strength endurance for the upper versus lower limbs across an adventure race (in which the lower limbs are utilised most heavily [121]); and (iii) the reduction in those functional capacities being much smaller than the reduction in exercise intensity of racing itself [121].

#### Neuroendocrine

Desensitisation to, or depletion of, stress-related hormones, humoral factors and neurotransmitters appears to have a role in the ‘selection’ of intensity during ultra-endurance exercise [92]. Research on prolonged, multi-day military training indicates that chronic elevation of circulating noradrenaline may lead to a desensitisation to the sympathetic response [7, 8, 77, 122, 123], which has even been observed within one bout of exercise (36–135 min at 5–10 % below anaerobic threshold [124]). Consistent with this, heart rate becomes lower despite a higher [noradrenaline]_plasma_ at submaximal exercise intensity following a 24-h adventure race simulation [125, 126]. Thus, perhaps the lower heart rate reflects a protective mechanism for the desensitisation, specifically of cardiac muscle.

#### Cardiovascular

On the other hand, cardiac dysfunction and ‘damage’ following ultra-endurance exercise has been reported repeatedly (reviewed in [127]). The adaptive desensitisation may reduce the pulse pressure and frequency and intensity of ventricular contractions, temporarily reducing work capacity and aiding homeostasis, while chronically, the prolonged and repeated myocardial loading is associated with functional and structural (mal)adaptations. Specifically, functional changes appear mostly reversible following 1 or 2 weeks of recovery [128, 129], while structural remodelling of the right ventricle and myocardial fibrosis in the interventricular septum is evident in some ultra-endurance athletes (e.g. [128]). Further, there is some suggestion that the potential for (mal)adaptive changes in cardiac tissue from prolonged exposure to exercise may explain the elevated prevalence of arrhythmias and sudden cardiac death in chronically fit athletes [130–133]. Although others [134] argue that the primary animal data that support this do not convincingly translate to the human setting, and the epidemiological data that provide the evidence for sudden cardiac death during marathon events do not distinguish the recreational from the elite athlete, nor do they account for potential pre-existing undiagnosed cardiac conditions that may have been brought on by the prolonged exercise [134].

In addition, Masters athletes with lifelong history of exercise training appear to have a blunted cerebrovascular response to arterial carbon dioxide content (PCO_2_) [135], which seems to conflict with the established link between impaired cerebrovascular responsiveness and disease [e.g. hypertension [136], diabetes [137], dementia [138]] and prediction of all-cause cardiovascular mortality [139]. Thomas and colleagues suggested that the blunted response they observed in their habitually fit Masters athletes was a consequence of the prolonged exposure to elevated arterial CO_2_ content from exercise (i.e. chronic adaptation), which would presumably include ultra-endurance forms. Finally, the peripheral vasculature may also show maladaptive responses to a prolonged history of ultra-endurance running, with recent reports showing lower large-artery compliance in runners than controls [140]. Collectively, there is limited direct evidence implying permanent cardiac, cerebrovascular or peripheral vascular damage after ultra-endurance exercise, acutely or chronically, although an inverted U- or J-shaped adaptive pattern may be present. Further work is needed to elucidate this area.

#### Cerebral

Understanding how the brain contributes to optimising performance in extreme environments has gained attention recently. Paulus and colleagues [141] showed that adventure racing athletes have altered (insular) cortex activation during an aversive interoceptive challenge consisting of increased respiratory effort. Interoception is a process suggested to be important for optimal performance because it links the perturbation of internal state as a result of external demands to goal-directed action that maintain a homeostatic balance [142]. Further, these findings in adventure racers were similar to the differential modulation of the right insular cortex in elite military personnel during combat-like performance [143]. Whether these differences in brain activation are a consequence of chronic adaptation or that individuals participating in these activities self-select into them, perhaps as a biological consequence of their neuroanatomy, remains to be determined. Nevertheless, Noakes’ premise [91] that sensory feedback to the brain, its integration and interpretation within the brain (as reflected in behavioural outcomes such as perceived exertion ratings or pace selection), with the interpretation potentially being adaptable, appears to be emerging as an important factor for optimal performance in extreme environments. Indeed, ‘brain endurance training’ for improved endurance performance may be an example of how the brain may adapt (see [144, 145]), and supports the role that the brain has in regulating power output. However, how effective brain training is within the context of extreme loading (e.g. adventure racing), which as already mentioned is often associated with severe sleep deprivation and energy deficits, is unknown. In addition, brain energetics have a likely role in performance within this context, since animal studies have shown that both exhaustive exercise [146, 147] and sleep deprivation [148] reduce brain glycogen stores. Matsui and colleagues have also illustrated that the brain adapts in a similar way to skeletal muscle following exercise, whereby brain glycogen is increased above basal levels following both exhaustive exercise and following 4 weeks of exercise training [147]. Interestingly, the areas of the brain that are most affected are the cortex and hippocampus, both involved in motor control and cognitive function.

Despite all of these findings, we still have limited understanding of the specific neuropsychophysiological processes under ultra-endurance conditions. With modern research methods and techniques becoming accessible in extreme loading settings (e.g. mobile MRI unit), the opportunity to improve this understanding is increasing, and such opportunity has provided new and unexpected insight. For example, MRI voxel-based morphometry (VBM) showed a volume reduction of about 6 % across the 2 months of TEFR in the brains of the ultra-endurance runners competing in that event [149]. As the normal age-related physiological brain volume reduction is less than 0.2 % per year [150, 151], these results appear to have significant implications. However, caution must be taken when interpreting these observations. The grey matter (GM) volume reduction observed was specific to distinct regions of the brain, and specifically regions normally associated with visuospatial and language tasks [152], which were likely to have received reduced activation during this repetitive and relatively isolated 2-month task. Interestingly, the energy-intensive default mode network of the brain also showed reductions in GM volume. However, given that 60–80 % of the brain’s high energy consumption is used in baseline activity [153], perhaps the resting state system is less important during such prolonged running, and the deactivation of this region serves a function of energy conservation during such a catabolic state [152]. Nevertheless, regardless of these acute brain composition changes observed during the TEFR, they all returned to pre-race volumes within eight months following the event. Further, these pre-race volumes were not different to a group of moderate-activity control participants, indicating no chronic (mal)adaptation from training for this event. Collectively, these structural brain data indicate that despite substantial changes to brain composition during the catabolic stress of an ultra-marathon, the observed differences seem to be reversible and adaptive.

A specific field of research is developing due to the recognition that evaluation of pain resilience and mental peculiarities of individuals who repeatedly survive ultra-endurance competitions unscathed can serve as a counter-model to pain and mental disorder research. Although the behaviour of the athletes with repetitive exhausting and painful training every day for several years might support the notion that they have better pain control, the results of Tesarz et al. [154] support the opposite interpretation. There seems to be similarities but also differences in the mechanisms of pain perception and pain control in endurance athletes compared to controls [118]. As discussions on physical and mental resilience to internal and external stimuli are growing [155], further investigations on personality traits in ultra-endurance athletes may become a relevant part in this new field of research.

#### Metabolic

The capacity of an individual to sustain exercise for prolonged periods of 100+ near-continuous hours or for many hours repeated over many days will depend partly on their capacity for endurance-related metabolism. Indeed, there is ample evidence illustrating metabolic adaptation to extreme loading scenarios. Increased fat oxidation has been reported from studies on polar expeditions [4, 5], although without an evident increase in the fat oxidative power of the sampled muscle, and a differential response for exercising muscles of the upper limb (increased fat oxidation) and lower limb (decreased fat oxidation) [4, 156]. Metabolic adaptations to an adventure race also reveal an extremely pronounced shift towards fat metabolism [68], as occurs also in multi-day military operations [157]. The shift to and reliance on fat metabolism for the predominately low-to-moderate exercise intensity associated with ultra-endurance exercise seem critical, as food intake can be restricted for a number of reasons such as carrying capacity and availability. Indeed, large energy deficits are evident in these settings [5, 14, 158, 159], illustrated well in the Stroud et al. study where both participants were virtually devoid of body fat (~2 %) and severely hypoglycaemic (0.3 mmol L^−1^) by the end of their 95-day Antarctica polar expedition [5].

#### Energy and nutrient stores

Energy expenditure can reach 70 MJ in a single 24-h exercise bout, but appears to be typically 30–45 MJ during multiday semi-continuous exercise (adventure racing; [14, 158]), or grand tour cycle races [160]. Consequently, and as mentioned above, there is a significant energy deficit typically observed within this setting, yet this appears not to result in hypoglycaemia [68]. The energy deficits lead to fat mass and lean mass loss, but this is regained when adequate recovery is allowed after the event [161, 162]. The homeostatic balance of micronutrients and trace elements is probably also compromised during prolonged continuous exercise; however, this remains unknown and possibly not of major importance within this time frame. Overall, performance and minimal energy (macronutrient) needs required to continue exercise until completion are determined by balancing the consumption of carbohydrates, the shift towards fat oxidation and the mode(s) and duration of exercise, as well as the combination of upper body vs lower body exercise.

## Conclusions

### Suggestions and future directions: Practice and research

In the present review, we have primarily focused on the upper end of load carriage and exercise tolerance and capacity. The acute musculoskeletal impacts of such loading are intuitive but the (mal)adaptations are less so. All physiological systems are impacted and these generally have strong capacity for adaptation. However, adaptation patterns of musculoskeletal and physiological systems are often U or J shaped and over time minimal or no load carriage will decrease one’s global load carrying capacity and eventually lead to severe adverse effects and manifest disease under minimal absolute but high relative loads. We advocate that further understanding of load carrying capacity and the inherent mechanisms leading to adverse effects may advantageously be studied in this perspective. Indeed, improved access to insightful and portable technologies is providing possibilities to explore these questions raised throughout the review.

As an industry, the need to impose regulations or restrictions for ultra-endurance competitions like adventure racing does not seem necessary, since evidence to date indicates that physiological feedback mechanisms and changes in perception of exertion and motivation as a consequence of sleep deprivation appear capable of protecting individuals from homeostatic failure. However, the net effect on ultra-endurance performance as well as the long-term health consequences of acute and chronic non-steroidal anti-inflammatory and analgesics drug use, often taken in combination with stimulants like caffeine during competition, does require clarification and understanding of how they may impact on this homeostatic control, and therefore athlete safety.

Finally, while humans have many intrinsic mechanisms to protect themselves against acute and to some extent chronic overloading, it is now clear that no such mechanisms exist to effectively protect against the numerous harmful impacts of chronic underloading. Hence, such guidelines or policy seem at least as important as any directed against overloading.

## Abbreviations

GI: gastrointestinal
Phys: physical
Physiol: physiological
MAP: mean arterial blood pressure
BRS: baroreflex sensitivity
PaCO_2_: partial pressure of arterial carbon dioxide
SCD: sudden cardiac death
CAD: cardiac arterial disease
MI: myocardial infarction
CBF: cerebral blood flow
PAD: peripheral arterial disease
TG: triglycerides
FIS: International Ski Federation
VO_2_ max: maximal oxygen consumption
MRI: magnetic resonance imaging
TEFR: TransEurope FootRace
Mq: musculus quadriceps
vl: vastus lateralis
vi: vastus intermedius
vm: vastus medialis
Mam: musculus adductor magnus
Msa: musculus sartorius
Mgr: musculus gracilis
Msm: musculus semimembranosus
Mst: musculus semitendinosus
Mbf: musculus biceps femoris
Cl: caput longum
Cb: caput brevis
PCO_2_: carbon dioxide
GM: grey matter
